# The clinical significance of intraoperative adverse events in laparoscopic radical hysterectomies for early-stage cervical cancer

**DOI:** 10.1186/s12905-023-02844-9

**Published:** 2024-01-02

**Authors:** Xiaolin Chen, Dongfang Lu, Yanmin Mu, Lingxiao Kong, Ling Zhang

**Affiliations:** Department of Gynecology, Xingtai Third Hospital, Xingtai, 054000 China

**Keywords:** Cervical cancer, Laparoscopic radical hysterectomy, Intraoperative adverse event, Surgical outcomes

## Abstract

**Objective:**

Surgical quality plays a vital role in the treatment of malignant tumors. We investigated the classification of intraoperative adverse events (iAE) (ClassIntra) in relation to the surgical quality control of laparoscopic radical hysterectomies.

**Methods:**

A prospective cohort of 195 patients who had undergone laparoscopic radical hysterectomies for early stage cervical cancer between July 2019 and July 2021 was enrolled. Participants were classified into either an iAE or non-iAE groups in accordance with their intraoperative status. Surgical outcomes, patient satisfaction, and quality of life were compared between the two groups.

**Results:**

Overall, 48 (24.6%) patients experienced 71 iAE. The iAE group was associated with significantly longer operative times (mean: 270 vs. 245 min, P < 0.001), greater blood loss (mean: 215 vs. 120 mL, P < 0.001), and longer postoperative hospital stays (median: 16 vs. 11 days). Larger tumors and poor technical performance significantly increased the risk of iAE (P < 0.05). Multivariate analysis revealed that iAE were the only independent risk factors for postoperative complications (hazard ratio, 15.100; 95% confidence interval: 4.735–48.158, P < 0.001). Moreover, patients who experienced iAE had significantly lower satisfaction scores and poorer quality of life (P < 0.05).

**Conclusions:**

ClassIntra may serve as an effective adjunctive tool for surgical quality control in laparoscopic radical hysterectomies.

**Supplementary Information:**

The online version contains supplementary material available at 10.1186/s12905-023-02844-9.

## Introduction

Cervical cancer is the fourth most common malignancy. It ranks fourth as the cause of death among women worldwide [1]. Surgery remains the cornerstone of early stage cervical cancer treatment [2]. Surgical outcomes, including adverse events (i.e., intra-, and post-operative complications), reflect surgical quality. Adverse events may, in turn, have an impact on survival outcomes [3, 4]. With an increase in surgical volume and the complexity of preoperative comorbidities, concerns regarding surgical adverse events have been continuously rising. Ongoing efforts have been made to standardize quality assessments and to promote quality improvement in cervical surgery.

In 2004, a classification system for postoperative complications (the Clavien–Dindo classification) was published [5]. This classification has been validated worldwide and has become a benchmark for reporting postoperative complications within and across researchers and institutions [6–8]. However, this system was not designed to assess intraoperative complications. Moreover, nearly half of surgical trials fail to report intraoperative complications because of the absence of a robust grading system [9]. Hence, the CLASSification of Intraoperative Complications (ClassIntra version 1.0) was constructed to grade intraoperative adverse events (iAE) by Dell-Kuster and colleagues [10]. This classification system evaluates any surgical or anesthetic iAE occurring between skin incision and closure, and can be incorporated into perioperative surgical safety checklists. A recent international study has demonstrated that an increasing grade of the most severe iAE was closely related to a more severe postoperative complication across various surgical fields [11]. However, this study included large amounts of patients who underwent gastrointestinal surgery (57%), while less than 2% of the patients underwent gynecological surgery. For the broader application of ClassIntra grade, further validations in the field of gynecology are warranted.

Since 1993, laparoscopic radical hysterectomies have been considered an alternative to open radical hysterectomies in patients with early stage cervical cancer [12]. However, the risk of intra- or post-operative complications may increase with this approach, owing to technical difficulties [3, 13, 14]. Uniform definitions of surgical adverse events are required to improve the safety evaluation of novel surgical techniques. Moreover, the introduction of surgical quality metrics is required for surgeons to overcome the early phase of the learning curve, for patients, and to reduce postoperative morbidity and mortality.

In this study, we analyzed the validity of the ClassIntra grade in patient who underwent laparoscopic radical hysterectomy for early stage cervical cancer and investigated the associations between iAE and surgical outcomes based on a prospective cohort. We also aimed to establish a reference for the application of laparoscopic radical hysterectomy in the treatment of cervical cancer.

## Materials and methods

### Study design and population

This is a retrospective cohort study based on a prospectively collected database. Consecutive patients with early stage cervical cancer who had undergone a laparoscopic radical hysterectomy at a tertiary referral teaching hospital between July 2019 and July 2021 were enrolled in this study and retrospectively reviewed. The study was approved by the Institutional Review Board of the Xingtai Third Hospital, and conducted in accordance with the Declaration of Helsinki (revised in 2013). This study followed the Strengthening the Reporting of Observational Studies in Epidemiology (STROBE) reporting guideline. Written informed consent was obtained from all participants.

### Inclusion and exclusion criteria

Patients who met the following criteria were included: (1) age > 18 years; (2) histologically confirmed primary cervical cancer, stages IA2 to IIA1 according to the International Federation of Obstetrics and Gynecology (FIGO) 2018; (3) no prior history of other malignancies; (4) no prior history of abdominal or pelvic radiotherapy; and (5) absence of severe diseases affecting vital organs. Those who had received neoadjuvant treatment or palliative surgery were excluded. Finally, 195 patients were enrolled.

### Treatment and follow-up

All patients underwent a laparoscopic radical hysterectomy, and surgical procedures were performed as previously reported [15]. Prior to this study, the surgeon involved had performed more than 200 surgeries. Perioperative care was provided in accordance with the recommendations of the Enhanced Recovery After Surgery (ERAS) Society recommendations [16]. After discharge, patients were followed up every three months for the first two years, every six months for the next three years, and annually thereafter.

### Data collection

Data on clinicopathological characteristics were obtained from a prospective database. Laboratory parameters, including white blood cell (WBC) count, hemoglobin (Hb) level, and albumin (Alb) level, were tested within one week before surgery and on the first, third, and fifth postoperative days for each patient. Postoperative complications were recorded and graded according to the Clavien-Dindo classification [5]. Patient satisfaction was measured on the day of discharge using the modified European Organization for Research and Treatment of Cancer (EORTC) IN-PATSAT14 scale on the discharge day [17]. A higher score indicated a higher level of satisfaction. One year postoperatively, quality of life (QOL) was measured using the EORTC Quality-of-Life-Core 30 (QLQ-C30). On the functional scales, a higher score indicates a better level of function; and on the symptom scales, a higher score indicates greater symptom severity [17].

### Outcome measurement

We defined iAE as any unexpected adverse event that occur from skin incision to skin closure, which included anaesthesia-related issues, excessive bleeding, and organ or tissue injury. ClassIntra (version 1.0) was derived by Dell-Kuster et al. who divided iAE into four grades according to severity and corresponding interventions [10, 11]. Additional details are presented in Table S1. All the patients were assessed using prospectively collected surgical records and videos. Two independent researchers (L.X.K. and L.Z.) who did not participate in the statistical analyses reviewed the unedited videos (repeated rewinds were permitted) and completed the confirmation form. In cases of disagreement, the iAE classification was resolved by consensus. Anesthesia status was determined using an electronic anesthesia system.

Objective Structured Assessment of Technical Skills (OSATS) was developed by Martin et al. and used to assess intraoperative technical skills [16]. The OSATS has seven specific scales, with points for each scale varying from one to five. A higher total score indicated better technical performance. The full rating scale is shown in Table S2. All the surgical procedures were scored by the same researchers using similar methods.

### Statistical analysis

The sample size calculation of this study was based on the assumption that the incidence of iAE would be 37% in gynecological surgery, with the expected postoperative complication rates of 48% and 28% in the iAE and non-iAE groups, respectively [11]. With a two-sided α of 5% and a power of 80%, a minimum sample size of 193 patients was required.

All statistical analyses were performed using the SPSS (version 23.0; IBM Corporation, Armonk, NY, USA) and R software version 4.1.2 (R Foundation for Statistical Computing, Vienna, Austria). Data were expressed as the median and interquartile range or mean and standard deviation (SD) for non-normally or normally distributed continuous variables, respectively, and as counts and percentages for categorical variables. Categorical variables were compared using the chi-square test or Fisher’s exact test. Continuous variables were compared using the t-test or Mann–Whitney U test. Univariate and multivariate analyses were performed using a logistic regression model; and hazard ratios (HRs) and 95% confidence intervals (CIs) were calculated. Statistical significance was set at a two-tailed *P*-value of < 0.05.

## Results

During the study period, 195 patients with FIGO stage IA2-IIA1 cervical cancer were treated with laparoscopic radical hysterectomies and included in this study. The general characteristics of the study participants are presented in Table 1.

**Table 1:** Baseline characteristics of the iAE and non-iAE groups

Characteristic	Overall (N = 195)	iAE group (N = 48)	Non-iAE group (N = 147)	*P* value
Age, years	47.6 ± 10.6	49.0 ± 9.4	47.2 ± 10.9	0.300
BMI, kg/m^2^				0.549
<25	160 (82.1)	38 (79.2)	122 (83.0)	
≥25	35 (17.9)	10 (20.8)	25 (17.0)	
FIGO stage				< 0.001
IA2	24 (12.3)	1 (2.1)	23 (15.6)	
IB1	131 (67.2)	28 (58.3)	103 (70.1)	
IB2	14 (7.2)	8 (16.7)	6 (4.1)	
IIA1	26 (13.3)	11 (22.9)	15 (10.2)	
Histological type				0.264
Squamous cell carcinoma	156 (80.0)	35 (72.9)	121 (82.3)	
Adenocarcinoma	35 (17.9)	11 (22.9)	24 (16.3)	
Adenosquamous carcinoma	4 (2.1)	2 (4.2)	2 (1.4)	
Tumor size, mm				< 0.001
<20	148 (75.9)	27 (56.3)	121 (82.3)	
≥20	47 (24.1)	21 (43.8)	26 (17.7)	
Depth of stromal invasion				< 0.001
≤1/2	131 (67.2)	22 (45.8)	109 (74.1)	
>1/2	64 (32.8)	26 (54.2)	38 (25.9)	
Lymphovascular space invasion				0.359
No	170 (87.2)	40 (83.3)	130 (88.4)	
Yes	25 (12.8)	8 (16.7)	17 (11.6)	
Lymph node metastasis				0.469
No	183 (93.8)	44 (91.7)	139 (94.6)	
Yes	12 (6.2)	4 (8.3)	8 (5.4)	
OSATS score	28.5 ± 1.6	27.2 ± 1.5	29.0 ± 1.5	< 0.001

Data were expressed as N(%) for categorical variables or mean ± standard derivation for normally distributed continuous variables

**Abbreviations**: iAE, intraoperative adverse event; BMI, body mass index; FIGO, International Federation of Gynecology and Obstetrics; OSATS, Objective Structured Assessment of Technical Skills

### Associations between iAE and patient characteristics

Of all the patients, 48 (24.6%) experienced 61 iAE (Table 2). The most severe iAE were grade I in 22 patients (11.3%), grade II in 17 patients (8.7%), grade III in seven patients (3.6%), and grade IV in two patients (1.0%). No grade V iAE occurred.

**Table 2:** Surgical outcomes of the iAE and non-iAE groups

Characteristic	iAE group (N = 48)	Non-iAE group (N = 147)	*P* value
Intraoperative outcomes			
Operation time, min	270 (248–291)	245 (222–270)	< 0.001
Blood loss, mL	215 (115–270)	120 (100–140)	< 0.001
Open conversion	2 (4.2)	0 (0.0)	0.060
Intraoperative complications			
Arrhythmia events	6 (12.5)	0	
Vessel injury	44 (91.7)	0	
Bladder injury	5 (10.4)	0	
Ureter injury	2 (4.2)	0	
Bowel injury	3 (6.3)	0	
Obturator nerve injury	1 (2.1)	0	
Postoperative complications	22 (45.8)	6 (4.1)	< 0.001
Haemorrhage	2 (4.2)	0 (0.0)	
Pelvic abscess including infected lymphocyst	4 (8.3)	0 (0.0)	
Urinary tract infection	7 (14.6)	4 (2.7)	
Ureteral stricture	3 (6.3)	0 (0.0)	
Urinary fistula	2 (4.2)	0 (0.0)	
Lymphatic leakage	1 (2.1)	2 (1.4)	
Ileus	1 (2.1)	0 (0.0)	
Wound complications	3 (6.3)	1 (0.7)	
Deep vein thrombosis	1 (2.1)	0 (0.0)	
Clavien-Dindo classification			0.549
I-II	18 (81.8)	6 (100.0)	
III-IV	4 (18.2)	0 (0.0)	
Postoperative recovery			
Return of bowel movement, days	3 (2–3)	2 (2–3)	0.713
Postoperative hospital stay, days	16 (11–21)	11 (10–13)	< 0.001
Blood transfusion	16 (33.3)	2 (1.4)	< 0.001

Data were expressed as N(%) for categorical variables, median (interquartile range) for non-normally distributed continuous variables, or mean ± standard derivation for normally distributed continuous variables

**Abbreviations**: iAE, intraoperative adverse event; OSATS, Objective Structured Assessment of Technical Skill

Compared to patients without iAE, those with iAE were at significantly more advanced FIGO stages (P < 0.001), had larger tumors (P < 0.001), deeper stromal invasion (P < 0.001), and lower OSATS scores (P < 0.001). No significant differences were found in age, body mass index (BMI), histological type, lymphovascular space invasion, or lymph node metastasis (P > 0.05, Table 1).

### Associations between iAE and surgical outcomes

Table 2 compares the surgical outcomes of the iAE and non-iAE groups. Patients in the iAE group experienced a significantly longer surgical time (mean: 270 vs. 245 min, P < 0.001) and greater blood loss (mean: 215 vs. 120 mL, P < 0.001). All open conversions occurred in two patients (4.2%) in the iAE group.

Postoperative complications were observed in 22 patients (45.8%) in the iAE group and in six patients (4.1%) in the non-iAE group, showing a statistically significant difference (P < 0.001). Severe postoperative complications occurred in four patients (18.2%) in the iAE group. The median postoperative hospital stay was significantly longer in the iAE group than in the non-iAE group (16 vs. 11 days, P < 0.001). Moreover, blood transfusions were significantly more frequent in the iAE group than in the non-iAE group (33.3% vs. 1.4%, P < 0.001).

**Fig. 1:**
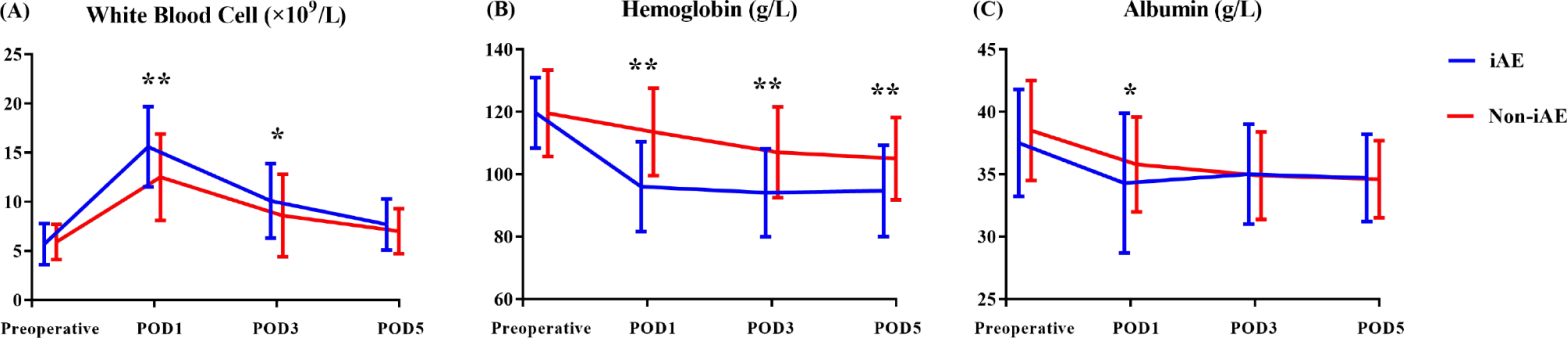
Laboratory findings including (**A**) white blood cell; (**B**) hemoglobin; and (**C**) albumin before surgery and on postoperative days 1, 3 and 5. *P < 0.05, **P < 0.01

As depicted in Fig. 1, the preoperative WBC count, HB level, and ALB level were comparable between the groups (P > 0.05). The HB levels were significantly lower in the iAE group than in the non-iAE group during the postoperative period (P < 0.05). In the iAE group, the WBC counts on postoperative days one and three were significantly higher, and the ALB levels on the first postoperative day were significantly lower.

### Univariate and multivariate analyses

The incidence of postoperative complications was significantly associated with a higher BMI, larger tumors, iAE, and lower OSATS scores (P < 0.05). Multivariate analysis showed that iAE was the only independent risk factors for postoperative complications (HR, 15.100; 95% CI:4.735–48.158, P < 0.001; Table 3). Moreover, factors independently affecting the incidence of iAE included tumor size (P = 0.035) and OSATS score (P < 0.001, Table S3).

**Table 3:** Univariate and multivariate analyses for postoperative complications

Characteristic	Univariate analysis		Multivariate analysis	
	HR (95% CI)	*P* value	HR (95% CI)	*P* value
Age, years		0.082		
<60	Reference			
≥60	2.272 (0.902–5.721)			
BMI, kg/m^2^		0.039		0.052
<25	Reference		Reference	
≥25	2.569 (1.048–6.297)		3.078 (0.991–9.562)	
FIGO stage		0.105		
IA2-IB1	Reference			
IB2-IIA1	2.078 (0.859–5.029)			
Histological type		0.476		
SCC	Reference			
Non-SCC	1.406 (0.550–3.593)			
Tumor size, mm		0.015		0.506
<20	Reference		Reference	
≥20	2.829 (1.226–6.527)		1.411 (0.511–3.894)	
Depth of stromal invasion		0.432		
≤1/2	Reference			
>1/2	1.392 (0.610–3.177)			
Lymphovascular space invasion		0.802		
No	Reference			
Yes	1.159 (0.366–3.671)			
Lymph node metastasis		0.066		
No	Reference			
Yes	3.312 (0.926–11.850)			
iAE		< 0.001		< 0.001
No	Reference		Reference	
Yes	19.885 (7.352–53.778)		15.100 (4.735–48.158)	
OSATS score	0.579 (0.437–0.767)	< 0.001	0.860 (0.619–1.194)	0.367

**Abbreviations**: HR, hazard ratio; CI, confidence interval; BMI, body mass index; FIGO, International Federation of Gynecology and Obstetrics; SCC, squamous cell carcinomai; iAE, intraoperative adverse event; OSATS, Objective Structured Assessment of Technical Skills

### Associations between iAE and patient satisfaction

Table 4 shows the results for patient satisfaction. Compared to the non-iAE group, the iAE group had a significantly lower overall quality rating score (mean: 69.0 vs. 78.4, P = 0.012). Regarding special items, the mean satisfaction scores for technical skill (72.3 vs. 78.9, P = 0.003) and comfort (60.3 vs. 73.6, P = 0.003) were significantly lower in the iAE group than in the non-iAE group.

**Table 4:** Patient satisfaction of the iAE and non-iAE groups

Scales	iAE group (N = 48)	Non-iAE group (N = 147)	*P* value
SATDTS	72.3 ± 15.2	78.9 ± 12.3	0.003
SATDIS	77.9 ± 14.5	79.1 ± 13.0	0.590
SATDIP	69.6 ± 13.4	70.2 ± 11.3	0.761
SATDAV	67.2 ± 18.1	68.1 ± 16.0	0.743
SATOTH	68.0 ± 17.9	69.5 ± 14.5	0.559
SATWAI	70.8 ± 13.7	71.3 ± 11.6	0.805
SATCSI	60.3 ± 22.8	73.6 ± 18.3	< 0.001
SATGEN	69.0 ± 24.5	78.4 ± 21.5	0.012

Data were expressed as mean ± standard deviation

**Abbreviations**: iAE, intraoperative adverse event; SATDTS, doctor’s technical skills; SATDIS, doctor’s interpersonal skills; SATDIP, doctor’s information provision; SATDAV, doctor’s availability; SATOTH, other personal interpersonal skills and information provision; SATWAI, waiting time; SATCSI, comfort special-item; SATGEN, overall quality rating

### Associations between iAE and QOL

At one year postoperatively, questionnaires were completed by 45 patients (93.8%) in the iAE group and 143 (97.3%) were in the non-iAE group (Table S4). The overall QOL score was significantly lower in the iAE group than in the non-iAE group (mean: 74.8 vs. 79.4, P < 0.001). The iAE group was associated with significantly worse role and emotional functioning than the non-iAE group (P < 0.05). Regarding the symptom scales, the iAE group experienced more significant problems with fatigue and pain than the non-iAE group (P < 0.05).

## Discussion

Surgical outcomes show a close correlation with the quality of intraoperative performance. In this study, we found that the incidence of iAE was significantly associated with perioperative outcomes. Patient-reported outcomes, including satisfaction and QOL, were significantly worse in the presence of iAE. To the best of our knowledge, this is the first study to verify the ClassIntra classification system for the evaluation of the quality of laparoscopic radical hysterectomies. This system may be a reliable tool to evaluate intraoperative surgical performance and guide postoperative care.

Surgery remains the cornerstone of the management of patients with early stage cervical cancer. Ensuring patient safety during the perioperative course has, therefore, been a topic of concern for surgeons [18, 19]. Given the close relationship between surgical performance and outcome, a robust grading tool is needed to evaluate surgeons’ performance in improving patients’ outcomes [20, 21]. The ClassIntra system is a standardized and comprehensive tool for assessing iAE across different surgical disciplines [10]. Unlike other classification systems [22–24], it has been validated on an international prospective cohort, based on a large sample size [11]. This system was used to identify and grade iAE during laparoscopic radical hysterectomies. The overall incidence of iAE was 24.6% in this study, which is similar to that reported by Liu (20%) [25] but lower than that reported by Dell-Kuster (37%) [11]. This difference may be explained by that all surgeries were performed by an experienced surgeon. Moreover, the incidence of iAE was higher than the rates of intraoperative complications reported by several randomized controlled trials [26], which could be explained by consideration regarding anesthesia events and our strict assessment.

Previous studies have attempted to address classification systems for iAE. For example, Francis et al. developed an EAES classification to evaluate iAE in laparoscopic surgery [24]. This classification also contained five grades but excluded anaesthesia-related adverse events. Kaafarani et al. proposed a 6-point severity classification system and demonstrated a significant association between severe iAE and postoperative complications [23]. However, this system was only analyzed in patients with accidental trauma. Unlike other systems, the ClassIntra classification system is the first comprehensive system that has been prospectively validated in an international, multicenter cohort involving any type of surgery [11]. Therefore, we adopted this classification system to assess iAE during laparoscopic radical hysterectomy due to its good generalizability.

It was found that the incidence of iAE was significantly associated with an increased risk of postoperative complications and prolonged hospital stay, as is consistent with the results of previous studies [10, 11, 25]. With the increasing application of innovative surgical techniques, decreasing intra- and post-operative adverse events has become even more important. In this regard, the ClassIntra grading system should be incorporated into routine practice so as to foreground patients with iAE and prevent further postoperative complications. Moreover, the OSATS score, which was designed to evaluate intraoperative technical performance, was closely related to postoperative complications. Although this score lost its independent value after adjusting for iAE, it was independently associated with the incidence of iAE, which was in line with previous findings [25]. The OSATS may assist in guiding surgical training and quality improvement interventions for less-experienced surgeons.

Regular collection of patient-reported outcomes can help improve patient-clinician communication, patient satisfaction, QOL, and overall survival [27, 28]. Given the negative impact of iAE on patient satisfaction and QOL, clinicians should enhance their medical monitoring of these “high-risk” patients during their hospitalizations and after discharge. Further research is required to address this issue.

Our study has several strengths. As the first study validating the efficacy of the ClassIntra classification system in the field of gynecological oncology, a prospective cohort was analyzed to reduce potential bias and improve the reliability. Additionally, this study was not only conducted to investigate the association between iAE and postoperative morbidity, but also explored the long-term impact of iAE on patient outcomes, thereby highlighting the need for individualized treatment and surveillance strategies in patients who experienced iAE.

This study has certain limitations. First, although all the data were derived from a prospective cohort, the study design was retrospective and selection bias was inevitable. Second, all patients were treated at a single institution in China, which may limit the generalizability of our findings. Finally, the ClassIntra grade was developed for all surgical disciplines and not specifically for cervical cancer surgery; therefore, specific items regarding this specialty should be included in the grading process.

In conclusion, iAE identified by the ClassIntra grade were significantly associated with postoperative complications and recovery as well as with patient satisfaction and QOL. This grade should be routinely applied in surgical quality control and clinical decision making, particularly in future clinical trials. For the broader application of ClassIntra grade in the field of gynecologic oncology, further studies will be performed in patients with other gynecologic malignancies.

### Electronic supplementary material

Below is the link to the electronic supplementary material.

Supplementary Material 1

Supplementary Material 2

## Data Availability

The datasets used and/or analyzed during the current study are available from the corresponding author on reasonable request.
