# Supplementary material for: The clinical significance of intraoperative adverse events in laparoscopic radical hysterectomies for early-stage cervical cancer
Source: BMC Womens Health. 2024 Jan 2;24:1. doi: 10.1186/s12905-023-02844-9 (PMC10763214; doi:10.1186/s12905-023-02844-9)
Supplement: Supplementary file 1 — Supplementary Material 1 [file 12905_2023_2844_MOESM1_ESM.docx]

**Table S1**. ClassIntra grade classification (version 1.0) of intraoperative adverse events.

| **Grade** | **Definition** | **Examples** |
| --- | --- | --- |
| Grade 0 | No deviation from the ideal intraoperative course | - |
| Grade I | Any deviation from the ideal intraoperative course: • Without the need for any additional treatment or intervention • Patient with no or mild symptoms | • Bleeding: bleeding above average from small calibre vessel, self-limiting or definitively manageable without additional treatment than routine coagulation • Injury: minimal serosal intestinal lesion, not requiring any additional treatment • Cautery: small burn of the skin, no treatment necessary • Arrhythmia: arrhythmia (eg, extrasystoles) without relevance |
| Grade II | Any deviation from the ideal intraoperative course: • With the need for any additional minor treatment or intervention • Patient with moderate symptoms, not life threatening, and not leading to permanent disability | • Bleeding: bleeding from medium calibre artery or vein, ligation; use of tranexamic acid • Injury: non-transmural intestinal lesion requiring suture(s) • Cautery: moderate burn requiring non-invasive wound care • Arrhythmia: arrhythmia requiring administration of antiarrhythmic drug, no haemodynamic effect |
| Grade III | Any deviation from the ideal intraoperative course: • With the need for any additional moderate treatment or intervention • Patient with severe symptoms, potentially life threatening or potentially leading to permanent disability | • Bleeding: bleeding from large calibre artery or vein with transient haemodynamic instability, ligation or suture; blood transfusion • Injury: transmural intestinal lesion requiring segmental resection • Cautery: severe burn requiring surgical debridement • Arrhythmia: arrhythmia requiring administration of antiarrhythmic drug, transient haemodynamic effect |
| Grade IV | Any deviation from the ideal intraoperative course: • With the need for any additional major and urgent treatment or intervention • Patient with life threatening symptoms or leading to permanent disability | • Bleeding: life threatening bleeding with splenectomy; massive blood transfusion; stay at intensive care unit • Injury: injury of central artery or vein requiring extended intestinal resection • Cautery: life threatening burn injury by cautery leading to fire requiring intensive care treatment • Arrhythmia: arrhythmia requiring electroconversion, defibrillation, or admission intensive care |
| Grade V | Any deviation from the ideal intraoperative course with intraoperative death of the patient | - |
| Note: These events were not defined as intraoperative adverse events: sequelae, failures of cure, events related to the underlying disease, incorrect site or incorrect patient surgery, or errors in indication. | | |

**Table S2**. Rating scale for the Objective Structured Assessment of Technical Skill.

| **Respect for tissue** | 1 | 2 | 3 | 4 | 5 |
| --- | --- | --- | --- | --- | --- |
|  | Frequently used unnecessary force on tissue or caused damage by inappropriate use of instruments. |  | Careful handling of tissue but occasionally caused inadvertent damage. |  | Consistently handled tissues appropriately with minimal damage. |
| **Time and motion** | 1 | 2 | 3 | 4 | 5 |
|  | Many unnecessary moves. |  | Efficient time/motion but some unnecessary moves. |  | Economy of movement and maximum efficiency. |
| **Instrument handling** | 1 | 2 | 3 | 4 | 5 |
|  | Repeatedly makes tentative or awkward moves with instruments. |  | Competent use of instruments although occasionally appeared stiff or awkward. |  | Fluid moves with instruments and no awkwardness. |
| **Knowledge of instruments** | 1 | 2 | 3 | 4 | 5 |
|  | Frequently asked for the wrong instrument or used an inappropriate instrument. |  | Knew the names of most instruments and used appropriate instrument for the task. |  | Obviously familiar with the instruments required and their names. |
| **Use of assistants** | 1 | 2 | 3 | 4 | 5 |
|  | Consistently placed assistants poorly or failed to use assistants. |  | Good use of assistants most of the time. |  | Strategically used assistant to the best advantage at all times. |
| **Flow of operation and forward planning** | 1 | 2 | 3 | 4 | 5 |
|  | Frequently stopped operating or needed to discuss next move. |  | Demonstrated ability for forward planning with steady progression of operative procedure. |  | Obviously planned course of operation with effortless flow from one move to the next. |
| **Knowledge of specific procedure** | 1 | 2 | 3 | 4 | 5 |
|  | Deficient knowledge. Needed specific instruction at most operative steps. |  | Knew all important aspects of the operation. |  | Demonstrated familiarity with all aspects of the operation. |

**Table S3**. Univariate and multivariate analyses for intraoperative adverse events..

| **Characteristic** | **HR (95% CI)** | **P value** |
| --- | --- | --- |
| FIGO stage (IB2-IIA1) | 0.891 (0.504-1.573) | 0.690 |
| Tumor size (≥20 mm) | 2.613 (1.067-6.395) | 0.035 |
| Depth of stromal invasion (>1/2) | 1.418 (0.523-3.846) | 0.493 |
| OSATS score (per 1 increment) | 0.451 (0.330-0.617) | <0.001 |
| **Abbreviations:** HR, hazard ratio; CI, confidence interval; FIGO, International Federation of Gynecology and Obstetrics; SCC, squamous cell carcinomai; OSATS, Objective Structured Assessment of Technical Skills. | | |

**Table S4**. Quality of life (QOL) of the iAE and non-iAE groups.

| **Items** | **iAE group (N = 45)** | **Non-iAE group (N = 143)** | **P value** |
| --- | --- | --- | --- |
| QLQ-C30 Functional scales |  |  |  |
| Global Health Status/QOL | 74.8 ± 6.5 | 79.4 ± 7.8 | <0.001 |
| Physical Functioning | 87.3 ± 5.6 | 88.8 ± 6.0 | 0.139 |
| Role Functioning | 83.0 ± 9.3 | 86.1 ± 6.9 | 0.017 |
| Emotional Functioning | 82.3 ± 12.2 | 87.5 ± 10.4 | 0.006 |
| Cognitive Functioning | 89.9 ± 7.3 | 90.1 ± 5.9 | 0.852 |
| Social Functioning | 80.5 ± 8.0 | 82.6 ± 7.3 | 0.102 |
| QLQ-C30 Symptom scales |  |  |  |
| Fatigue | 15.2 ± 6.4 | 11.3 ± 4.0 | <0.001 |
| Nausea & Vomiting | 3.8 ± 2.6 | 3.7 ± 1.4 | 0.740 |
| Pain | 18.2 ± 7.0 | 15.6 ± 5.5 | 0.011 |
| Dyspnea | 3.0 ± 2.2 | 2.8 ± 1.7 | 0.523 |
| Insomnia | 12.8 ± 8.1 | 10.6 ± 6.3 | 0.059 |
| Appetite loss | 9.0 ± 4.2 | 9.2 ± 2.7 | 0.708 |
| Constipation | 15.6 ± 6.6 | 14.7 ± 4.8 | 0.320 |
| Diarrhea | 4.6 ± 2.7 | 4.2 ± 1.9 | 0.270 |
| Financial difculties | 37.8 ± 14.6 | 33.0 ± 18.1 | 0.107 |
| Data were expressed as mean ± standard deviation. **Abbreviations:** iAE, intraoperative adverse event; QLQ-C30, Quality-of-Life-Core 30**.** | | | |
